# The importance of feedback for medical students’ development

**DOI:** 10.2147/AMEP.S134419

**Published:** 2017-03-22

**Authors:** Rahul Rajesh Amin, Rohin Patel, Prashant Bamania

**Affiliations:** Faculty of Medicine, Imperial College London, Kensington, London, United Kingdom

**Dear editor**

We read with great interest the study by McKenzie et al,^1^ which explores the impact of providing feedback to students on their ability to carry out a clinical procedure. This study was particularly interesting to us as fifth-year medical students at Imperial College London who have learnt several clinical skills during rotations. A noteworthy finding was that education involving providing feedback to students had a positive impact on their performance.^1^ Furthermore, the recommendation that clinical skills training be accompanied with feedback resonated strongly with us.

During our first clinical year, we had regular teaching sessions about clinical skills including venepuncture, cannulation, and suturing. Despite being encouraged to engage in self-assessment, we found this was less effective than external feedback. Ward et al^2^ showed the limitations of self-assessment in creating improvements; however, Srinivasan et al^3^ concluded that feedback was pivotal in improving self-assessment. We practiced the skills we learnt on simulation dummies. Here, we were individually supervised by clinical teaching fellows who provided us with detailed, student-specific feedback and constructive criticism of our performance, allowing us to better reflect on our own abilities and where improvements can be made and implemented. The timeliness and level of student-specific feedback we received motivated us to keep working on the deficiencies identified. Over time this all helped improve our abilities to perform clinical skills. This provided a strong platform from which we could further enhance our skills through practice in real clinical scenarios. Doctors and nurses supervised us, provided feedback, and advocated changes so we became more competent and confident in our abilities, but importantly, we also learnt how to build strong rapport with patients and make them as comfortable as possible.

In some rotations of our second clinical year, we often ran consultations under doctor supervision. After consultations, doctors provided us with personalized feedback about how we could improve which we could take away and put into practice. We learnt crucial questions to ask during history taking, ways of building patient rapport, and tips on becoming more competent at examinations. Feedback is given and stored on a common app on student’s individual iPads. This is useful for future reference because we can easily see where we went wrong before, and exactly how we can improve. Ashby et al^4^ concluded that electronic feedback can enhance both the quantity and quality of the feedback itself. The feedback received was invaluable and helped boost our confidence in our own abilities.

Thus, we consider feedback an important learning tool for students. We agree that providing student’s feedback can improve their abilities to perform clinical skills^1^ and also take histories, perform examinations, and tend to patient comfort. We suggest that electronic feedback be implemented in all forms of medical training. Hence, students can easily access and store feedback for future reference and remind themselves of what went well before and what could be improved. We feel that providing students regular feedback about their clinical performances will be invaluable to their development as competent doctors.
