# Supplementary material for: The importance of feedback for medical students’ development
Source: Adv Med Educ Pract. 2017 Mar 22;8:249–51. doi: 10.2147/AMEP.S134419 (PMC5367734; doi:10.2147/AMEP.S134419)
Supplement: Related texts

# Authors’ reply

**Published:** 2017-03-22

**Authors:** Susan McKenzie, Annette Burgess, Craig Mellis

**Affiliations:** 1 Central Clinical School; 2 Education Office, Sydney Medical School, The University of Sydney, Sydney, NSW, Australia

**Dear editor**

Thank you for the opportunity to respond to the letter by Dr Amin and colleagues.

We wish to thank Dr Amin and colleagues for their interest in our recent article^1^ and have found their feedback encouraging for our work.

We find the use of electronic feedback to be of particular interest as we are currently developing a similar electronic application for the use of students’ “smart phones”. We anticipate by capturing feedback in this manner, students have a great opportunity to reflect on their procedural skills performance, and to be able to improve their skills when necessary.

## References

[1] McKenzie S, Burgess A, Mellis C (2017). Interns reflect: the effect of formative assessment with feedback during pre-internship. Adv Med Educ Pract.
